# Hospital admissions and mortality for STEMI and NSTEMI during COVID-19 outbreak: a meta-analysis: Paolo Matteo Angeletti

**DOI:** 10.1093/eurpub/ckac131.206

**Published:** 2022-10-25

**Authors:** E Altobelli, PM Angeletti, F Marzi, F D'Ascenzo, R Petrocelli, G Patti

**Affiliations:** 1 Department of Life, Public Health and Environmental Sciences, University of L’Aquila, L'Aquila, Italy; 2 Cardiac Surgical Intensive Care Unit, Giuseppe Mazzini Hospital, Teramo, Italy; 3 Cardiovascular and Thoracic Department, Division of Cardiology, University of Turin, Turin, Italy; 4 San Timoteo Hospital, ASREM Molise, Termoli, Italy; 5 Department of Translational Medicine, Maggiore della Carità Hospital, University of Eastern Piedmont, Novara, Italy

## Abstract

**Background:**

During SARS-CoV-2 pandemic, various studies have shown a significant reduction of Emergency Department (ED) presentations for acute cardiac diseases requiring in-hospital management. The aim of our study was to quantify hospital admission and mortality, comparing pandemic period and pre-pandemic period in different countries.

**Methods:**

We performed an updated meta-analysis of observational studies to quantify on a large basis the impact of the SARS-CoV-2 outbreak on patients admitted to the ED for STEMI and NSTEMI. The literature research was conducted on PubMed, EMBASE, Scopus, Science Direct, Web of Science and Cochrane database registry on 6 January 2022. We performed a random-effect model meta-analysis.

**Results:**

A total of 61 studies were included: came from Italy, China, Germany, Israel, Turkey, France, Helvetic Confederation, India, Poland, Spain, US, UK, Albania, Austria, Egypt, Greece, Iran, Ireland, Japan, Pakistan, Portugal, Saudi Arabia and Canada. Hospital admissions for STEMI decreased in most country. The countries with the high levels of reduction were Italy (IRR = 0.68) and Germany (IRR = 0.69). Mortality rates for STEMI increased differently among countries analyzed: p = 0.003. The highest mortality rate was in Serbia (OR = 2.15), followed by Italy (OR = 1.97), Pakistan (OR = 1.69) and France (OR = 1.55). Among the High-Income countries, the highest mortality rate was in Italy (OR = 3.71), the highest among the Upper-Middle-Income was in Serbia (OR = 2.15) and the highest among Low- Middle-Income was in Pakistan (OR = 1.69). Regarding NSTEMI, hospital admissions showed that Italy had the lowest value for with IRR = 0.59. Among countries, the meta-regression subgroups analysis, showed statistical difference (p < 0.001).

**Conclusions:**

Our meta-analysis may represent a robust snapshot that might help healthcare systems manage and assist an expected higher number of people coming to the hospitals for severe, post-acute cardiological issues in the future.

**Key messages:**

• The study shows hospital admission and mortality, comparing pandemic period and pre-pandemic period in different countries.

• Epidemiological data suggests that one-fourth to one-third of MI patients, in large areas of the globe, during the COVID-19 pandemic in 2020, remained at home and did not have access to ED.

